# Management Patterns and Outcomes of Ogilvie Syndrome in the Absence of Standardized Protocols: A Single-Center Case Series From Puerto Rico

**DOI:** 10.7759/cureus.117197

**Published:** 2026-09-30

**Authors:** Gustavo Alayón-Rosario, Manuel Frau-Conde, Gabriela Santiago-Casanova, Marcia Alayón-Rosario

**Affiliations:** 1 Medicine - Surgery and Gastroenterology, Ponce Health Sciences University, Ponce, PRI; 2 Medicine - Surgery and Gastroenterology, University of Medicine and Health Sciences, Basseterre, KNA; 3 Surgery, Ponce Health Sciences University, Ponce, PRI

**Keywords:** acpo, acute colonic pseudo-obstruction, colonic dilation, colonoscopic decompression, healthcare limitations, neostigmine therapy, ogilvie syndrome, postoperative complications, resource-limited setting, surgical intervention

## Abstract

Purpose: Ogilvie syndrome, or acute colonic pseudo-obstruction (ACPO), is an uncommon, potentially life-threatening condition characterized by acute colonic dilation without mechanical obstruction. In Puerto Rico, limited access to standardized treatment pathways and gastroenterology services may complicate management in resource-limited settings. This study aimed to describe the clinical characteristics, management patterns, and inpatient outcomes of bedridden patients with ACPO treated at a secondary hospital in Puerto Rico.

Case description: This retrospective observational case series included six consecutive adult patients who met the diagnostic criteria for ACPO between 2020 and 2024. All patients were bedridden and had multiple comorbidities accompanied by electrolyte abnormalities. Symptoms were present for a mean of 7.2 days before intervention. Standard conservative management consists of correction of electrolyte abnormalities, nasogastric and rectal tube decompression, neostigmine therapy, and colonoscopic decompression. Surgical intervention was performed in patients who failed conservative management or were considered at imminent risk of perforation. At this institution, where no standardized ACPO protocol was in place, surgical intervention was performed in 66.7% (n = 4), and postoperative complications occurred in 16.7% (n = 1). Colonoscopic decompression was not performed in 66.7% (n = 4), and neostigmine was not administered in 83.3% (n = 5). One patient who received comprehensive conservative management recovered without surgery.

Conclusion: This case series documents inconsistent use of guideline-recommended conservative therapies alongside a high frequency of surgical intervention. Because of the retrospective design, small sample size, and absence of a comparison group, the study cannot establish that protocol availability or treatment patterns caused the observed surgical or mortality outcomes. The findings support consideration of standardized ACPO management pathways and further prospective evaluation in resource-limited settings.

## Introduction

Ogilvie syndrome, also known as acute colonic pseudo-obstruction (ACPO), is characterized by acute colonic dilation in the absence of mechanical obstruction. ACPO is uncommon, with an estimated incidence of approximately 100 cases per 100,000 inpatient admissions [1]. Delayed recognition can lead to ischemia or perforation; mortality is approximately 15% in uncomplicated cases and may reach 30%-44% when these complications occur [2].

The pathophysiology is not fully understood but is thought to involve autonomic nervous system dysregulation. Reported risk factors include advanced age, hospitalization, immobility, recent surgery or trauma, electrolyte abnormalities, serious underlying comorbidities, and medications that impair gastrointestinal motility, including opioids, anticholinergics, neuroleptics, tricyclic antidepressants, and calcium channel blockers [1,3].

Diagnosis is clinical and radiologic by exclusion, requiring demonstration of colonic dilation without a mechanical obstructing lesion. Initial management includes bowel rest, nasogastric decompression, correction of fluid and electrolyte abnormalities, mobilization when possible, and discontinuation of medications that may worsen colonic stasis. When supportive measures are unsuccessful, neostigmine therapy or colonoscopic decompression may be used [3-5]. Surgical intervention is reserved for ischemia, perforation, peritonitis, or disease that remains refractory to nonoperative management [3,5].

Published evidence describing ACPO management in Puerto Rico remains limited. Given the reported shortage of specialist services in Puerto Rico [6], we hypothesized that limited access to gastroenterology services and inconsistent application of guideline-recommended conservative measures may be associated with delayed escalation of care and greater reliance on surgical intervention, although this has not been formally established. The primary aim was to characterize the clinical presentation, use of guideline-recommended conservative therapies, and inpatient outcomes, including surgical intervention, among bedridden patients with ACPO at a secondary hospital in Puerto Rico. A secondary aim was to inform the development of standardized conservative management protocols for ACPO within the Puerto Rican healthcare context.

## Materials and methods

Ethical considerations

All patient-identifying information was omitted to protect the privacy and confidentiality of the patients. Cases were selected using prespecified inclusion and exclusion criteria. This study was approved by the Institutional Review Board (#2405199073) of Ponce Research Institute.

Data collection

This retrospective observational case series included adults older than 18 years who were diagnosed with ACPO at a secondary hospital in Puerto Rico between January 1, 2020, and December 31, 2024. The study site was an approximately 140-bed acute-care hospital with emergency and intensive-care capabilities. Gastroenterology services were available; however, the medical record specifically documented a lack of emergency gastroenterology on-call coverage in at least one case. To identify potentially eligible cases for retrospective review, the electronic medical record was queried using ICD-10 code K59.81 for colonic pseudo-obstruction. All K59.81-coded cases during the study period were reviewed consecutively, and no additional case selection was performed beyond the prespecified inclusion and exclusion criteria. Inclusion required acute colonic dilation demonstrated on imaging without a mechanical obstructing lesion. Exclusion criteria were age younger than 18 years, mechanical bowel obstruction confirmed by imaging, or incomplete or missing medical records. ACPO was diagnosed clinically and radiologically by exclusion. Abdominal CT was the preferred imaging modality to characterize colonic dilation and exclude mechanical obstruction (Figures 1, 2). Six patients met the study criteria. Records were deidentified before analysis. Retrospectively collected variables included demographic characteristics, comorbidities, past medical and surgical history, presenting symptoms and examination findings, duration of symptoms before intervention, bedridden status, electrolyte abnormalities, recent surgery, immobility, and routine medications.

**Figure 1:**
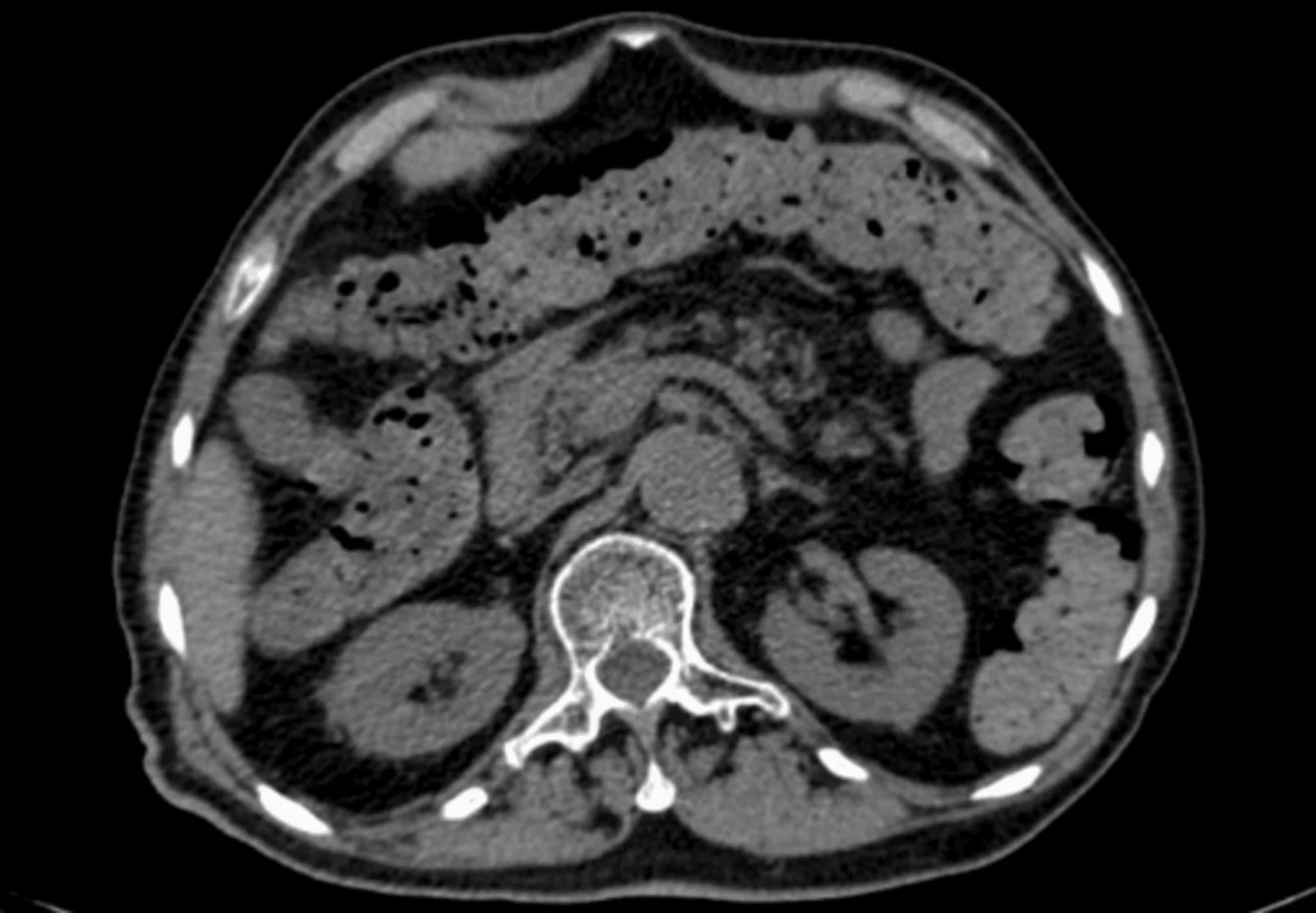
Axial CT scan of the colon before Ogilvie syndrome.

**Figure 2:**
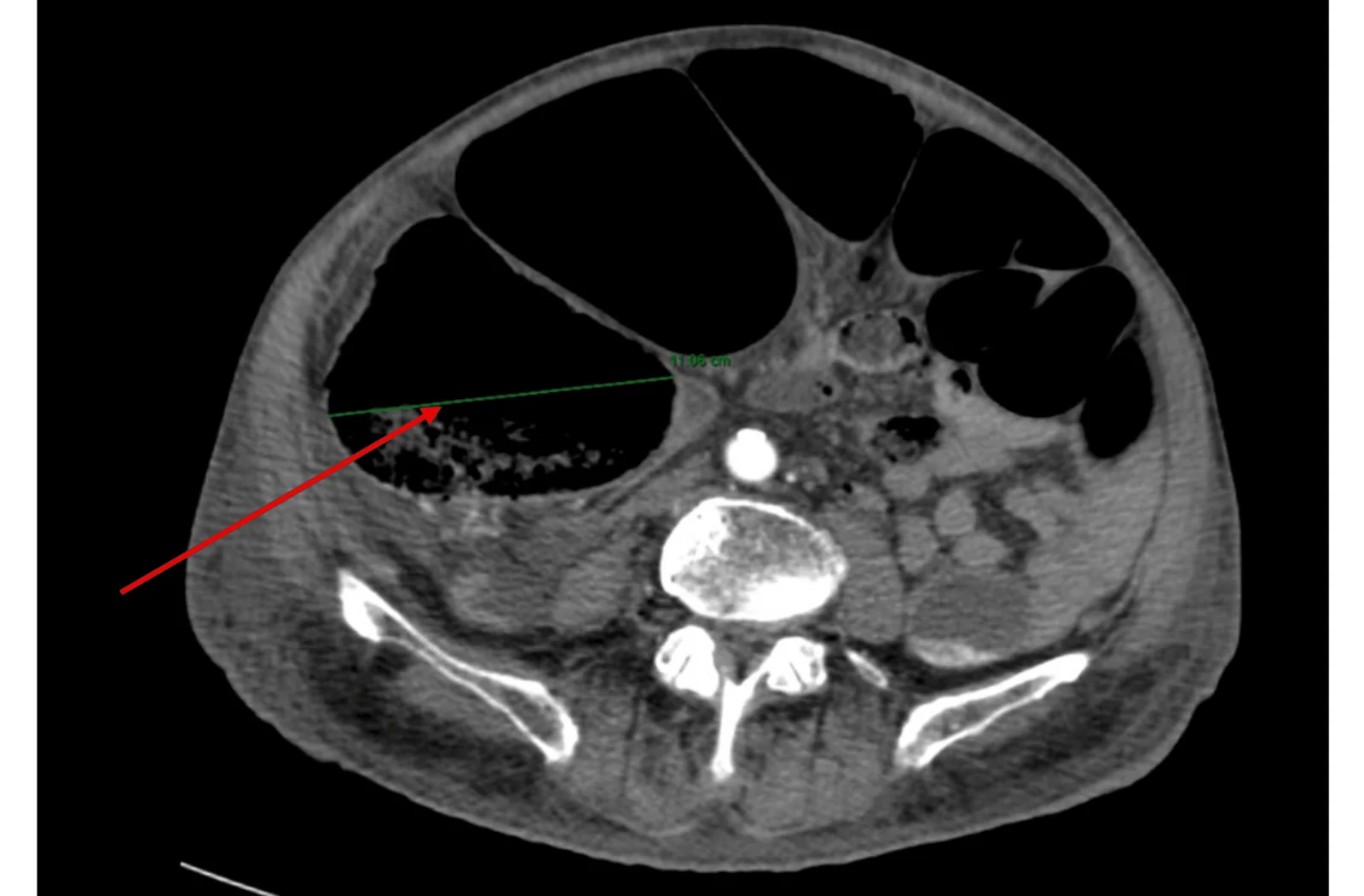
Axial CT scan of the colon with Ogilvie syndrome. The arrow points at area of largest colon diameter of the patient with 11.06 cm.

Diagnostic data included CT findings and available complete blood count, arterial blood gas, and complete metabolic panel results. Treatment data included supportive measures, nasogastric decompression, electrolyte correction, neostigmine administration, colonoscopic decompression, and surgical intervention. Surgical management consisted of total abdominal colectomy with end ileostomy.

The primary outcomes were symptom resolution and reduction of colonic dilation after treatment. Secondary outcomes included correction of electrolyte abnormalities, intraoperative complications, postoperative complications, in-hospital mortality, and length of hospital stay. For this retrospective analysis, successful nonoperative management was defined as documented resolution of ACPO during the index hospitalization without progression to surgical intervention. A colonoscopic decompression attempt was recorded when the procedure was documented as performed, and procedure-level response was abstracted from the treating team’s postprocedure documentation. Patients who demonstrated a transient response to conservative, pharmacologic, or endoscopic therapy but subsequently required surgery were not classified as successful nonoperative management outcomes.

Surgical approach

Patients who failed conservative management or were considered at imminent risk of perforation underwent total abdominal colectomy with end ileostomy through a midline laparotomy using standard operative technique. No intraoperative complications occurred in this series.

Data analysis

The study was limited to descriptive statistical methods to analyze demographic, clinical, radiologic, and laboratory findings. Categorical variables were reported as frequencies and percentages, while continuous variables were expressed as means ± standard deviations (SDs) and ranges. Cases with incomplete medical records were excluded from analysis.

Data were compiled and analyzed using Microsoft Excel (Microsoft Corp., Redmond, WA, USA). Efforts to address potential bias included consecutive review of all K59.81-coded cases during the study period, application of prespecified inclusion and exclusion criteria, and use of objective outcome definitions, including radiographic assessment of colonic distension. No inferential statistical analysis was performed because of the small sample size and descriptive study design.

## Results

As presented in Table 1, the mean age of patients was 78.3 (range 51-90). The gender distribution was equal, with three men (50%) and three women (50%). All six patients were bedridden. The mean body mass index (BMI) was 23.3 ± 2.8 (range: 20.1-27.3). Half of the patients (50%, n = 3) fell within the normal BMI range (18.5-24.9), while the remaining 50% (n = 3) were classified as overweight (BMI 25.0-29.9). None of the patients were underweight or obese. The patients had multiple comorbidities, with cardiovascular disease being present in 83.3% (n = 5) of patients, diabetes mellitus type II in 83.3% (n = 5), hypertension in 66.7% (n = 4), and hypothyroidism in 50% (n = 3) of cases (Table 1). Less common comorbidities include history of stroke in 33.3% (n = 2), dementia in 33.3% (n = 2), autism, hypercholesterolemia, and a recent myocardial infarct in 16.7% of patients (n = 1). There were various types of cardiovascular diseases among the patients in this study, including hypertrophic cardiomyopathy, atrial fibrillation, chronic arrhythmia, coronary artery calcifications, and atherosclerosis of the abdominal aorta. One patient exhibited all of these. The clinical presentation demonstrated classic symptoms associated with Ogilvie syndrome (Table 2). Findings included abdominal distension and constipation in 100% of patients (n = 6). Abdominal pain was present in 83.3% of cases (n = 5), while flatus was reported in 50% of patients (n = 3). Abnormal bowel sounds were present in all six patients, with 83.3% of patients experiencing tympanic sound (n = 5), and one patient experiencing hypoactive sound (16.7%). Less common symptoms included nausea and vomiting in 33.3% of cases (n = 2), and diarrhea, gastroesophageal reflux disease (GERD), hemiparesis, dysphagia, and paraplegia in 16.7% of patients (n = 1).

**Table 1:** Study group characteristics. SD: standard deviation; BMI: body mass index

Demographics	N (%)
Age	6
Mean ± SD	78.3 ± 12.8 years
Median ± SD	82.0 ± 12.8 years
Range (min-max)	51-90
BMI (n)	6
Mean ± SD	23.3 ± 2.8
Median ± SD	23.1 ± 2.8
Range (min-max)	20.1-27.3
Sex	6 (100.0)
Male	3 (50.0)
Female	3 (50.0)
BMI categories	6 (100.0)
<18.5	0 (0)
18.5-24.9	3 (50)
25.0-29.9	3 (50)
>30.0	0 (0)
Comorbidities	6 (100.0)
Cardiovascular disease	5 (83.3)
Diabetes mellitus II	5 (83.3)
Hypertension	4 (66.7)
Hypothyroidism	3 (50)
Stroke	2 (33.3)
Dementia	2 (33.3)
Autism	1 (16.7)
Hypercholesterolemia	1 (16.7)
Myocardial infarct	1 (16.7)

**Table 2:** Study group symptoms. GERD: gastroesophageal reflux disease

Presenting symptoms	N (%)
Abdominal distension	6 (100.0)
Constipation	6 (100.0)
Abdominal pain	5 (83.3)
Flatus	3 (50)
Nausea	2 (33.3)
Vomiting	2 (33.3)
Diarrhea	1 (16.7)
GERD	1 (16.7)
Hemiparesis	1 (16.7)
Dysphagia	1 (16.7)
Paraplegia	1 (16.7)
Type of bowel sound	6 (100.0)
Tympanic	5 (83.3)
Hypoactive	1 (16.7)

As shown in Table 3, laboratory results showed significant electrolyte imbalances, especially hypokalemia, which was present in 66.7% of patients (n = 4). The mean serum potassium level was 2.9 ± 0.6 mEq/L, which indicates moderate to severe hypokalemia in most patients. Among the six patients, 33.3% (n = 2) had hyperchloremia and 33.3% (n = 2) had hypochloremia (Table 4). None of the patients exhibited hyperkalemia, hypernatremia, hyponatremia, hypercalcemia, hypermagnesemia, or hyperphosphatemia. Table 4 shows the mean serum sodium was 139.3 ± 2.4 mEq/L, and the mean chloride was 102.7 ± 6.1 mEq/L. Four out of the five available white blood cell counts were elevated, with a mean of 13.6 ± 3.6 x 10³/µL (range 9.4-18.7). The mean blood urea nitrogen (BUN) was 29.7 mg/dL (range: 6-65). Four patients (66.7%) had a history of recent surgery.

**Table 3:** Metabolic state disorders.

Metabolic state	N (%)
Hypokalemia	6 (100.0)
Yes	4 (66.7)
No	2 (33.3)
Hypochloremia	6 (100.0)
Yes	2 (33.3)
No	4 (66.7)
Hyperchloremia	6 (100.0)
Yes	2 (33.3)
No	4 (66.7)

**Table 4:** Study group metabolic lab values. SD: standard deviation; BUN: blood urea nitrogen

Lab values	Normal range
Na^+^	135-145 (mEq/L)
Mean ± SD	139.3 ± 2.4
Median ± SD	138.0 ± 2.4
Range (min-max)	137-144
Cl^-^	98-106 (mEq/L)
Mean ± SD	102.7 ± 6.1
Median ± SD	103.6 ± 6.1
Range (min-max)	93-110
K^+^	3.5-5 (mEq/L)
Mean ± SD	2.9 ± 0.6
Median ± SD	2.7 ± 0.6
Range (min-max)	2.3-3.91
BUN	6-24 (mg/dL)
Mean ± SD	29.7 ± 21.5
Median ± SD	27.0 ± 21.5
Range (min-max)	6-65

As presented in Table 5, the mean time from symptom to intervention was 7.2 ± 5.1 days. The mean length of hospital stay was 23.5 days (range: 1-66 days). Imaging showed colonic dilation in all patients and confirmed the diagnosis by excluding mechanical obstruction. CT scans were the preferred imaging modality for evaluating all six patients. Axial CT imaging highlights the markedly distended colon characteristics of Ogilvie syndrome (Figure 2), while coronal views further illustrate the extent of severity and distribution of colonic dilation across segments (Figure 3). The average diameter of the most dilated bowel segment was 14.3 ± 4.6 cm (range: 9.6-21.0 cm). Medication history revealed wide variation; apixaban was the only medication taken by more than one patient (33.3%, n = 2). Other medications included vancomycin, antipsychotics (e.g., aripiprazole and risperidone), anticonvulsants (e.g., lamotrigine and topiramate), antidepressants (e.g., sertraline), and medications for chronic disease management (e.g., enalapril, metformin, alprazolam, aspirin, omeprazole, and famotidine).

**Table 5:** Study group intervention characteristics. SD: standard deviation; NGT: nasogastric tube

Clinical variables	N (%)
Symptoms prior to intervention	6
Mean ± SD	7.2 ± 5.1 days
Median ± SD	5.5 ± 5.1
Range (min-max)	1-14 days
Length of stay	6
Mean ± SD	23.5 ± 21.0 days
Median ± SD	19.0 ± 21.0 days
Range (min-max)	1-66 days
Diameter of bowel	6
Mean ± SD	14.3 ± 4.6 cm
Median ± SD	12.7 ± 4.2 cm
Range (min-max)	9.6-21 cm
Perforation	6 (100.0)
Yes	0 (0.0)
No	6 (100.0)
Ischemia	6 (100.0)
Yes	1 (16.7)
No	5 (83.3)
Intraoperative blood loss	4
Mean ± SD	66.3 ± 58.2 mL
Median ± SD	50.0 ± 58.2 mL
Range (min-max)	15-150 mL
Bedridden	6 (100.0)
Yes	6 (100.0)
No	0 (100.0)
Type of intervention	6 (100.0)
Conservative	6 (100.0)
Surgical intervention	4 (66.7)
NGT decompression	6 (100.0)
Yes	6 (100.0)
No	0 (100.0)
Colonoscopic decompression	6 (100.0)
Yes	2 (33.3)
No	4 (66.7)
Neostigmine treatment	6 (100.0)
Yes	1 (16.7)
No	5 (83.3)
Surgery	6 (100.0)
Yes	4 (66.7)
No	2 (33.3)
Surgical patients evaluated for complications	4
Intraoperative complications	0 (0.0)
Postoperative complications	1 (16.7)
Vital state	6 (100.0)
Alive	4 (66.7)
Deceased	2 (33.3)

**Figure 3:**
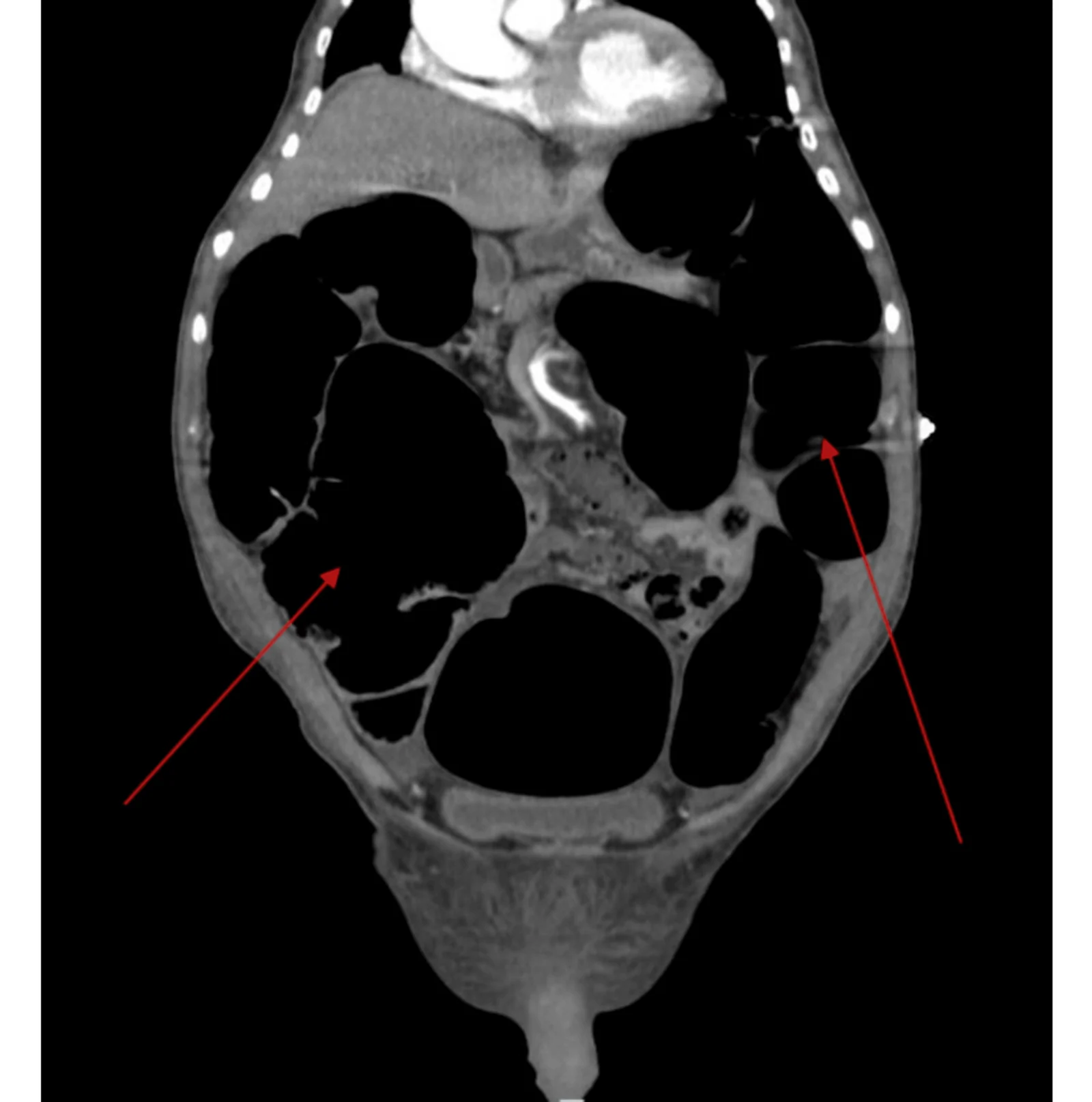
Coronal CT scan view of the colon with Ogilvie syndrome. Arrows point to areas of large bowel dilatation.

Management

All patients received conservative management, including supportive measures and nasogastric decompression (Table 5). Neostigmine was administered in one case (16.7%). Colonoscopic decompression was performed in 33.3% of patients (n = 2). A greater proportion of patients required surgical intervention, with 66.7% (n = 4) undergoing operative management. All surgical procedures consisted of total abdominal colectomy with end ileostomy.

Postoperative complications occurred in one patient (16.7%), consisting of abdominal wall gangrene at the ileostomy site. No intraoperative complications were reported. There were no cases of colonic perforation, and only one patient (16.7%) exhibited signs of ischemia. The mean intraoperative blood loss was 66.3 mL (range 15-150 mL) (Table 5).

Outcomes and mortality

The overall mortality rate in this series was 33.3% (n = 2). One of the four surgical patients died from postoperative sepsis (surgical mortality, 25%), and one nonsurgical patient died from cardiac arrest before electrolyte correction could be initiated. The four surviving patients were discharged; three had undergone surgery, and one had recovered with nonoperative management. Follow-up data beyond discharge were unavailable.

## Discussion

This case series describes a discrepancy between guideline-recommended stepwise management of ACPO and the treatment patterns observed in this cohort, particularly the limited use of neostigmine and colonoscopic decompression before surgical intervention. The cohort was characterized by advanced age, immobility, multiple comorbidities, and medication exposures that are recognized in the literature as factors associated with ACPO [1,3]. This observation is in accordance with the clinical practice guidelines of the American Society of Colon and Rectal Surgeons, which emphasize that ACPO most frequently affects patients who are older, hospitalized, or institutionalized with significant comorbidities, immobility, or recovering from surgery or trauma [6]. The most consistent initial findings across patients with ACPO included marked colonic dilation, varying degrees of electrolyte imbalance, and diverse comorbidities (a variety of cardiovascular diseases in 83.3% and hypothyroidism in 50%) recognized in the literature [1,7] as plausible contributors to Ogilvie syndrome, each representing well-established factors associated with colonic dysmotility.

Medication exposure was another potential contributing factor in this cohort. Several patients were receiving medications that may affect gastrointestinal motility. Because this descriptive case series did not evaluate medication causality, these exposures should be interpreted as possible contributors rather than confirmed causes of ACPO [7]. Further studies are needed to evaluate these associations in larger Puerto Rican cohorts.

The mean time from symptom onset to intervention was 7.2 ± 5.1 days. Current guidelines recommend 48-72 hours of supportive management before escalation when there is no clinical improvement [3,5]. The observed interval exceeded this window; however, because symptom timing was obtained retrospectively and the study had no comparison group, the present series cannot determine whether this interval reduced the likelihood of successful conservative management. Specific reasons for the observed treatment interval were not systematically documented in the retrospective records; therefore, the study cannot determine whether delayed recognition, patient-related factors, transfer processes, or specialty availability accounted for the interval.

Prolonged colonic dilation increases wall tension, particularly when the cecal diameter exceeds 12 cm, and is associated with greater risk of ischemia and perforation [3,7]. The mean diameter of the most dilated bowel segment in this cohort was 14.3 ± 4.6 cm (range: 9.6-21.0 cm). Prolonged symptom duration and marked dilation were observed alongside a 16.7% postoperative complication rate and 33.3% mortality; however, this small retrospective series does not permit a causal relationship to be established.

Structural and logistical limitations within Puerto Rico's healthcare system may affect timely access to ACPO management [6]; however, this study was not designed to measure these system-level effects. In this cohort, limited use of guideline-recommended therapies occurred alongside a documented instance in which emergency gastroenterology on-call coverage was unavailable. In most cases, conservative therapy consisted of supportive measures and nasogastric decompression, and four patients subsequently underwent surgery.

Neostigmine was administered to one patient, with subsequent documentation of two bowel movements and return of bowel sounds. Despite this initial clinical response, the patient ultimately required surgical intervention and therefore was not considered a successful nonoperative management outcome. The available retrospective record did not specify the neostigmine dose, route, administration protocol, or monitoring details. Guideline-based pharmacologic therapy with neostigmine is recommended when supportive management fails in the absence of contraindications and requires cardiac monitoring with atropine immediately available because of the risk of bradycardia [3,5,8]. Reasons for nonadministration of neostigmine in the remaining five patients were not consistently documented. Colonoscopic decompression was performed in two patients, with successful decompression documented in one. In at least one case, the medical record stated that no gastroenterologist was on call for emergent colonic decompression; patient-specific reasons for nonperformance in the other cases were not consistently documented. This 50% observed response rate is descriptive, and the small sample and retrospective design do not permit attribution of differences from published success rates to treatment timing, bowel diameter, or resource availability.

Four of the six patients in this series (66.7%) underwent total abdominal colectomy with end ileostomy. This proportion should not be directly equated with rates from dissimilar study populations. In the national inpatient analysis by Ross et al., 6.3% of patients underwent surgery alone and 0.5% underwent both colonoscopy and surgery [9]. In the selected single-center cohort reported by Joechle et al., 35% underwent upfront surgery, while 38% of patients initially managed conservatively and 32% initially managed endoscopically required escalation [10]. These differences are descriptive and may reflect variation in case selection, disease severity, treatment definitions, and healthcare setting. Ross et al. reported a 15.9% procedural complication rate across the entire national cohort rather than a surgery-specific postoperative complication rate [9].

The observed 33.3% mortality occurred in a medically complex cohort, including one death from postoperative sepsis and one cardiac arrest before electrolyte correction could be initiated. Vanek and Al-Salti emphasized that mortality among surgically treated patients with ACPO may reflect the severity of underlying illness [11]. Ross et al. similarly reported increasing morbidity and mortality with increasing procedure invasiveness, likely reflecting greater illness severity [9]. Delayed intervention, electrolyte abnormalities, comorbid illness, and limited specialty access were present in this cohort, but the study design does not establish that any of these factors caused the observed deaths.

Despite offering meaningful insight into the clinical management of Ogilvie syndrome, this case series has several limitations. All K59.81-coded cases were reviewed consecutively, but the retrospective design remains vulnerable to documentation and measurement bias. In addition, the absence of a comparison group precludes causal inference regarding the relationships among protocol availability, treatment patterns, surgical intervention, and mortality. All cases were managed within a secondary hospital setting with limited access to advanced gastroenterological services, critical care monitoring, and multidisciplinary support. As such, the treatment patterns and outcomes observed may not fully represent those in tertiary-care centers with greater resources and subspecialist availability. The small sample and single-center setting also limit generalizability. Given the sample size of six patients, percentages are presented descriptively with accompanying patient counts and should not be interpreted as population-level estimates. Comparisons with surgical or mortality rates reported in other studies are contextual only and were not subjected to inferential statistical testing.

Taken together, these findings support prioritizing timely gastroenterology access, availability of neostigmine with appropriate monitoring, and provider training in guideline-based escalation of care in resource-limited settings [3,5]. Because this series was descriptive and lacked a comparison group, it cannot determine whether these measures would reduce surgery or mortality. Prospective evaluation of a standardized ACPO management pathway is needed.

## Conclusions

This case series describes inconsistent use of guideline-recommended conservative therapies and a high frequency of surgical intervention among six bedridden patients with ACPO treated at a secondary hospital in Puerto Rico. Because of the retrospective design, small sample size, and absence of a comparison group, the study cannot determine whether the lack of standardized protocols caused the observed surgical or mortality outcomes. The findings support consideration of standardized, evidence-based ACPO management pathways, expanded provider education, and further prospective evaluation in resource-constrained settings.
